# 

**Published:** 1922-08

**Authors:** 


### RECENT APPOINTMENTS.

### Public Health.

Dr. Mary Turner, of Manchester, to be Assistant School Medical Officer for Crewe.

Dr. Kenneth McAlpine, R.A.M.C. and late Assistant Medical Officer for Birmingham, to be Assistant School Medical Officer for Stoke-on-Trent.

Dr. Dawson Robertson, late Asst. Medical Officer of Health for Willesden, to be Medical Officer of Health for Boston, in succession to Dr. Arthur Tuxford, resigned.

Dr. Andrew Gilmour, M.D., M.R.C.P.E., D.P.H., late M.O.H., &c., for Hindley U.D.C., to be Medical Officer of Health and School Medical Officer for the Borough of Wimbledon. Dr. Gilmour held a commission in the R.A.M.C. from 1914 to 1919.

Mr. John Theodore Macnab, M.A., M.B., B.Ch., M.R.C.S., L.R.C.P., D.P.H., late Assistant M.O.H. for the Shropshire County Council, to be Medical Officer of Health for the Borough of Stafford. During the war Mr. Macnab served as a surgeon in the Royal Navy.

### Institutional.

Dr. Eric Pritchard to be Consulting Physician to the Queen's Hospital for Children.

Mr. James McKay, Acting Secretary of the Hospital for Sick Children, Great Ormond Street, W.C.1, to be Secretary.

Dr. W. G. Bendle, late Assistant Medical Superintendent, to be Medical Superintendent of Paddington Hospital.

Dr. A. Archdale, of Cambridge, to be Medical Superintendent of Sunderland Borough Mental Hospital.

Mr. W. H. Leake, M.B., Ch.B.(Leeds), to be Resident Aural Officer at Leeds General Infirmary.

Mr. L. R. Broster, M.S., F.R.C.S., to be Assistant Surgeon to the Queen's Hospital for Children, Hackney Road, E. 2.

Dr. G. W. Lloyd, Assistant Medical Officer at Camberwell Infirmary, to succeed Dr. R. W. Wilson as Medical Superintendent at Croydon Infirmary and Union House.

Professor Alexander Miles Kennedy, M.D., who holds the chair of medicine at the Welsh National Medical School, Cardiff, has been elected Dean in succession to Professor David Hepburn, C.M.G., M.D.

Dr. Henry J. C. Gibson, Edinburgh, has been appointed Medical Superintendent of Dundee Royal Infirmary, in succession to the late Dr. H. E. Fraser. After a distinguished career at Glasgow and Edinburgh Universities, and various administrative appointments in war hospitals, Dr. Gibson became medical superintendent of Craiglockhart Hospital.

Miss Holmes has been appointed Secretary-Collector to the Royal Bucks Hospital, Aylesbury, in succession to Captain Illingworth Butler. Miss Holmes had been for fifteen years on the staff of the Royal Waterloo Hospital for Women and Children, where she became corresponding secretary before taking a similar post at Liverpool. Her qualifications outweighed the preference for a man, the claims of ex-Service men, and the argument from unemployment. There were 350 applications, including half-a-dozen from women.

### PARLIAMENTARY QUESTIONS AND ANSWERS.

July 3.—Sir Alfred Mond (to Mr. Foot). The number of insured persons in England and Wales entitled to medical benefit is estimated at approximately 13,250,000. The number of names on the lists of insurance practitioners and approved institutions is slightly over 12,800,000. The number of insured persons who have not selected a doctor is on this basis 450,000, or 3½ per cent.; but in consequence of the difficulty of keeping any precise record of the movements of the insured population there is inevitably some inflation in the doctors' lists, and the actual number of unassigned persons exceeds this figure. But the vast majority of unassigned persons are those who neglect to select a doctor until they are in need of treatment, and who then have a right to immediate treatment, although not at the time on any doctor's list. Thus the panel practitioners collectively are at risk in respect of them. The number of insured persons who do not avail themselves of their right to medical benefit when they require treatment is believed to be a relatively insignificant and diminishing fraction. The remainder of the question is based upon a misapprehension. Under the agreement with the doctors, there is no such division of the practitioners' fund as the hon. member suggests. The total sum available in any area is based upon the total number of insured persons for whose treatment, irrespective of the numbers assigned or unassigned, the practitioners in the area are collectively responsible. The available statistics as to the work done by insurance practitioners do not distinguish between patients who were or were not on the list of a practitioner at the time treatment was given.

July 5.—Sir J. Gilmour (to Mr. T. Thomson). The annual reports for 1920 of Medical Officers of Health for 1,500 local authorities showed that out of 1,085,000 houses inspected during the year 24,210 were reported as unfit. Information as to the number of persons inhabiting these houses is not available.

Sir A. Mond (to Mr. Lunn). The death rates in the case of zymotic diseases, or of cancer, for each decennial period from 1851 to 1910, so far as regards England and Wales, will be found in Table 10 of Part III. of the Supplement to the 75th Annual Report of the Registrar-General, which was published in 1919. The figures for the last decennium, 1911 to 1920, have not yet been published, but the uncorrected death rates (per million living) during that period are as follows: Small-pox, 0; measles, 275; scarlet fever, 47; whooping cough 184; diphtheria, 141; enteric fever, 35; diarrhœal diseases, 603; cancer, 1,117. These rates are subject to revision.

July 10.—Sir Alfred Mond (to Mr. Hurd). I shall be glad to consider any statements by medical authorities which my hon. friend can send me in support of the conclusion he mentions, both as to an alarming increase of cancer and as to its association with tinned foods chemically prepared and of foreign origin. I am always prepared to consult experts other than those associated with my Department, but, as at present advised, I do not think that there is need for the appointment of a special committee to investigate this subject.

Sir Alfred Mond (to Mr. Hurd). I am informed that the amount of condensed separated or skimmed milk imported during the first five months of this year was about 503,000 cwt., as compared with about 285,000 cwt. of condensed whole milk. Representations have been received on the question of issuing regulations as to labelling. This matter is under my consideration, and I shall be happy to consider any suggestions on the point. I have no power to alter the present statutory requirement that condensed separated milk must be labelled as "machine-skimmed" milk.

Sir Alfred Mond (to Lieut.-Col. Hurst). It has been decided to revoke the regulation under which the Registrar-General has hitherto declined to register births of children who have attained the age of 7 years, thus removing any limitation as to the period within which an application may be made. [A public announcement has since been made by the Registrar-General to this effect.]

July 11.—Sir Alfred Mond (to Mr. Hurd). I do not propose to insert any provision in the Milk Bill to deal with condensed milk, but I am considering what can be done under existing powers to protect the public against any misconceptions as to the nature of the article sold as condensed milk.

Sir John Baird (to Mr. T. Thomson). Out of every 20s. of the total amount received from all sources into the National Health Insurance Fund it is estimated that 12s. 2¼d. goes in benefit, 5s. 1¾d. to reserve and 2s. 8d. on administration. The corresponding figures for the Unemployment Fund are 17s. 10d. in benefit and 2s. 2d. in administration (including the cost of dealing with insured workpeople at Unemployment Exchanges).

### PROGRESS OF LEGISLATION.

The Audit (Local Authorities, &c.) Bill has received the Royal Assent.

The National Health Insurance Bill and the Local Government and Other Officers Superannuation Bill has passed through Committee in the House of Lords with amendments.

The Milk and Dairies (Amendment) Bill has been sent from the House of Lords to the House of Commons.

A Smoke Abatement Bill has been introduced in the House of Lords on behalf of the Government. The proposals in the Bill are no doubt designed to stimulate public discussion on the subject, but there does not appear to be any likelihood that it will become law this Session.

A short Bill introduced in the House of Commons to enable women to sit upon Visiting Committees, a very useful proposal, has made no further progress.

---

The Women's Holiday Fund, 76 Denison House, 296 Vauxhall Bridge Road, S.W.1, appeals for help to provide seaside or country holidays for poor London housewives and working women. A failure of health or nerves is often averted by a timely rest, with good food and good air. A fortnight's holiday, including railway fare, costs about £3, or £3 10s. for a mother and baby, the women contributing what they can.

The London offices of Lord Mayor Treloar's Cripples' Homes (Alton) have been moved to 25 Ely Place, E.C.1, a house in that quiet enclosure behind tall gateways off Holborn Hill. Ely Place was once part of the garden surrounding the town house of the Bishops of Ely, when as Lords of Parliament they came up to London. As such it was a "Libertie" and "Sanctuary," which still enjoys some of the autonomy it possessed in the past. It is governed by Commissioners elected by the householders. The Commissioners levy the rates and pay the watchman, who, in his ancient uniform, closes the gates every evening and "calls the hours" throughout the night, as did his predecessors.

---

Talcum-Cleaver is a pleasantly scented toilet powder, well adapted for nursery use and notably fine in grain. It is sterilised during manufacture and packed in handy refillable tins with pierced screw-tops, which ensure that the powder remains uncontaminated while in use. The manufacturers are Messrs. F. S. Cleaver & Sons, Ltd., of Twickenham, Middlesex.

At the 44th annual general meeting of the "Sanitas" Company, Ltd., the Chairman, Mr. C. T. Kingzett, F.I.C., F.C.S., said that, having regard to the depressed state of trade in the country, the great lack of export business, and the necessity of further writing down the value of stocks in view of fallen prices, the company had done as well as could be expected in the year under review. The turnover had amounted to little short of the best years of its history. The profit on the year's trading sufficed to pay the full dividend of 9 per cent. on the preference shares and 4½ per cent. on the ordinary shares. They had been able, recently, to reduce the retail prices of their chief retail articles, "Sanitas Fluid" and "Sanitas Powder," and it was gratifying to know that the sales of "Sanitas Fluid" in bottles had exceeded in number those of any previous year. A great deal of irresponsible matter was published from time to time on the subject of disinfection, and emphasis had been laid upon the alleged impossibility, or difficulty, of disinfecting infected rooms; but his own personal investigations had demonstrated that infected apartments containing furniture, fittings, carpets and even pictures, could be effectually disinfected by the use of "Sanitas Formigators" or "Sanitas Sulphur Candles."

### OFFICIAL REPORTS RECEIVED.

**(Year to Dec. 31, 1921, unless otherwise stated.) :—**

Queen Victoria's Jubilee Institute for Nurses.
Zenana Bible and Medical Mission.
Barking Urban District Council: Report of the M.O.H. and Sanitary Inspector.
The Ranyard Nurses, 25 Russell Square, W.C.1.
The Overseas Nursing Association. Year to March 31, 1922.
Central Council for Infant and Child Welfare. Year to March 31, 1922.
National Council for the Unmarried Mother and her Child. Fourth Annual Report, 1922. 4d.
County Borough of Blackburn. Annual Report by Dr. W. Allen Daley, Medical Officer of Health.
City of Lincoln Urban Sanitary Authority. Report of the Medical Officer of Health.

**Hospitals and Homes.**

Guy's Hospital.
Bradford Royal Infirmary.
General Hospital, Nottingham.
Adelaide Hospital, South Australia.
James Murray's Royal Asylum, Perth.
Royal Sussex County Hospital, Brighton.
David Lewis Northern Hospital, Liverpool.
Tunbridge Wells Homœpathic Hospital and Dispensary.
Royal Infirmary of Edinburgh. Year to October 1, 1921.
Worcestershire Mental Hospital, Barnsley Hall, Bromsgrove.
James Murray's Royal Asylum, Perth. Year to March 31, 1922.
Lebanon Hospital for Mental Diseases, Asfuriyeh, Beyrout, Syria. Year to March 31, 1922.
Western Counties Institution, Starcross, near Exeter (for Mental Defectives). Year to March 31, 1922.
Down District Lunatic Asylum, Downpatrick. Statistical Returns for the year 1921; Financial Returns for the year to March 31, 1922.
Spelthorne S. Mary and S. Bridget's, Bedfont, near Feltham, Middlesex. (For women who have fallen into habits of intemperance by misuse of drugs or alcohol.)
Worksop General Dispensary and Victoria Hospital, year to May 31, 1922.

